# *Quercus salicina* Blume Inhibits Bone Loss via Dual Effects on Osteoblasts and Osteoclasts

**DOI:** 10.3390/nu18213578

**Published:** 2026-10-29

**Authors:** Suhyun Cho, Nadzeya Lazerka, Soyeon Hong, Minji Gwon, Byeong Jun Jeon, Jeong Do Kim, Gyhye Yoo

**Affiliations:** 1 Gangneung Science & Industrial Promotion Agency, Gangneung 25440, Republic of Korea; 2 Smart Farm Research Center, Gangneung Institute of Natural Products, Korea Institute of Science and Technology (KIST), Gangneung 25451, Republic of Korea; 3 Division of Natural Product Applied Science, KIST School, University of Science and Technology (UST), Seoul 02792, Republic of Korea; 4 Department of Plant Science, Kangwon National University, Gangneung 25457, Republic of Korea

**Keywords:** osteoporosis, *Quercus salicina* Blume, osteogenic activity, anti-osteoclastic activity

## Abstract

**Background/Objectives**: Osteoporosis is a progressive metabolic bone disorder with reduced bone mass and microarchitectural deterioration, leading to an increased risk of fractures. With an aging population, there is a growing demand for safe and effective long-term therapeutic methods for osteoporosis and medicinal plants could be promising candidates as novel agents for osteoporosis. In this study, we investigated the effects of the 70% ethanol extract of *Quercus salicina* Blume (QS), an herbal plant, on osteoporosis. **Methods**: MC3T3-E1 mouse pre-osteoblast cells and RAW264.7 mouse macrophage cells were utilized to assess osteoblast and osteoclast differentiation, respectively. QSEE (10 mg/kg and 40 mg/kg) was orally administered for 12 weeks in OVX mice, a postmenopausal osteoporosis animal model, and bone parameters were evaluated using Micro-Computed Tomography (Micro-CT). Ex vivo experiments were conducted using primary cells isolated from mouse femurs and tibias. Expression levels of proteins and mRNAs related to osteoblasts and osteoclasts were estimated by Western blot and Quantitative Real-Time PCR (qRT-PCR). **Results**: QSEE showed osteogenic activity by increasing alkaline phosphatase (ALP) activity, protein and mRNA expression related to osteoblast differentiation. QSEE decreased osteoclast differentiation by suppressing tartrate-resistant acid phosphatase (TRAP) activity, protein and mRNA expression related to osteoclast differentiation. In OVX mice, QSEE administration restored bone microarchitecture decreased by OVX. In primary cells isolated from mouse femurs, QSEE increased osteoblast-related markers and decreased osteoclast-related markers. **Conclusions**: These findings suggest *Quercus salicina* Blume has potential as an anti-osteoporosis agent by inducing osteoblast differentiation and suppressing osteoclast differentiation.

## 1. Introduction

Osteoporosis is a metabolic bone disease characterized by bone loss and fragility fractures. As the world population ages, osteoporosis is emerging as a significant public health issue that needs to be addressed [1]. Bones maintain their structural stability through the process of bone remodeling, which is a continuous interaction between the formation by osteoblasts and the resorption by osteoclasts [2]. When this balance is disrupted due to various factors, osteoporosis occurs. Therefore, targeting the balance between osteoblasts and osteoclasts may provide an effective approach for osteoporosis therapy. However, there is currently no treatment that completely cures osteoporosis, and existing treatments such as bisphosphonates and denosumab are accompanied by severe side effects such as atypical fractures, cardiovascular risk and rebound bone loss after treatment discontinuation [3]. For these reasons, there is a need to find new alternative treatments that can overcome the limitations of current treatments [4].

Osteoblasts are bone-forming cells derived from mesenchymal stem cells that are responsible for bone mineralization and maturation [5]. Osteoblast differentiation is regulated by key signaling pathways such as Transforming growth factor β (TGF-β), Bone morphogenetic protein (BMP) and Wnt/β-catenin [6,7,8]. These signaling pathways regulate the expression of Runt-related transcription factor 2 (RUNX2), a major transcription factor for osteoblast differentiation [9]. Osterix (OSX) is a downstream target of RUNX2 and regulates early osteoblast formation [10]. RUNX2 and OSX promote the expression of osteoblast and osteogenic markers such as alkaline phosphatase (ALP), Collagen type 1 (COL1A1), Osteocalcin (OCN), and Osteopontin (OPN), resulting in osteoblast activation and bone formation.

Osteoclasts are bone-resorbing cells derived from hemopoietic progenitors of the macrophage lineage [11]. The binding of RANKL to its receptor, RANK, induces the activation and differentiation of osteoclasts. This binding activates various signaling pathways such as MAPK, PI3K/Akt, and NF-κB [12]. These signaling pathways induce the expression of the master transcription factor Nuclear factor of activated T cells (NFATc1), a main transcription factor in osteoclast differentiation [13,14]. NFATc1 functions together with c-Fos to promote the expression of osteoclast-related genes such as tartrate-resistant acid phosphatase (TRAP), Cathepsin K (CTSK), dendritic cell-specific transmembrane protein (DC-STAMP) and matrix metalloproteinase-9 (MMP9).

*Quercus salicina* Blume (QS) is a traditional medicinal herb that grows naturally in Korea and Japan. QS is known to have anti-inflammatory, anti-diabetic, anti-bacterial, antioxidant, and anti-allergic properties [15,16,17,18]. Many studies have reported the presence of large amounts of phenols and high antioxidant activity in QS extracts [15,17]. Studies have identified numerous bioactive constituents in the leaves of QS, including a wide range of phenolic acids (e.g., gallic acid, ellagic acid) and flavonoids (e.g., quercetin, kaempferol) as well as tannins. Despite these known biological activities of QS, its effects in restoring bone health remain unknown. We hypothesize that QS can be a potential treatment for osteoporosis. In this study, we investigated the effect of the 70% ethanol extract of QS (QSEE) on osteoblast and osteoclast differentiation, as well as measured bone parameters in an OVX mouse model administered with QSEE.

## 2. Materials and Methods

### 2.1. Plant Materials and Extraction

QS (*Quercus salicina* Blume) was purchased from Herbmaul (Cheongju-si, Chungcheongbuk-do, Republic of Korea). Dried plants (50 g) were extracted with 70% ethanol for three days (72 h) at room temperature with occasional agitation. QS ethanol extract (QSEE) was filtered through filter paper. The filtrate was concentrated using the SpeedVac SPD2030 Integrated System Kit (SPD2010; Thermo Scientific, MA, USA) until the solvent evaporated. The total extract weight was 0.21 g (extraction yield 0.415 ± 0.04%).

### 2.2. Cell Culture for Osteoblast and Osteoclast Differentiation

The mouse pre-osteoblast cell line MC3T3-E1 Subclone 4 (ATCC, Manassas, VA, USA) was cultured in alpha minimum essential medium (α-MEM; Welgene, Gyeongsan-si, Gyeongsangbuk-do, Republic of Korea) containing 10% fetal bovine serum (FBS, Gibco, Grand Island, NY, USA) and 1% penicillin–streptomycin (P/S, Hyclone, Logan, UT, USA) in a humidified 5% CO_2_ incubator at 37 °C. For osteoblast differentiation, the culture medium was replaced with a differentiation medium (DM) consisting of α-MEM supplemented with 10 mM β-glycerophosphate (Sigma-Aldrich, St. Louis, MO, USA) and 50 µg/mL L-ascorbic acid (Sigma-Aldrich).

The mouse macrophage cell line RAW 264.7 (ATCC) was cultured in Dulbecco’s Modified Eagle Medium (DMEM; Welgene) containing 10% FBS (Gibco) and 1% P/S (Hyclone) under the same incubator conditions. For osteoclast differentiation, RAW 264.7 cells were seeded and allowed to adhere for 24 h, after which the DMEM was replaced with α-MEM supplemented with 50 ng/mL recombinant murine RANKL (Peprotech, Cranbury, NJ, USA).

### 2.3. ALP Activity

MC3T3-E1 cells were seeded at 2.5 × 10^4^ cells/well in a 24-well plate and treated with QSEE, dissolved in dimethyl sulfoxide (DMSO). Cells were treated with 20 or 40 µg/mL of QSEE under DM. Twenty-four hours after seeding, cells were treated with QSEE for 7 days. Cells were co-treated with QSEE and MSAB (MCE, Monmouth Junction, NJ, USA), SB-431542 (MCE) and Noggin (Thermo Scientific, MA, USA) as inhibitors of the Wnt/β-catenin, TGF-β and BMP pathways, respectively. Cells were lysed with 0.1% Triton X-100 lysis buffer, and cell lysates were reacted with 10 mM 4-Nitrophenol solution (SensoLyte pNPP Alkaline Phosphatase Assay Kit, AnaSpec, Fremont, CA, USA) at 37 °C for 30 min. The reaction was stopped with 0.2 M NaOH and alkaline phosphatase (ALP) activity was measured by the absorbance at 405 nm using a multi-detection microplate reader (Synergy HT, BioTek Instruments, Winooski, VT, USA).

### 2.4. TRAP Activity

RAW 264.7 cells were seeded at 1 × 10^4^ cells/well in a 24-well plate and incubated for 24 h. The medium was then replaced with osteoclast differentiation medium containing QSEE (40 or 80 µg/mL) or vehicle for 4 days. After differentiation, the medium was removed, and cells were gently washed with Dulbecco’s phosphate-buffered saline (DPBS). For the TRAP activity assay, cells were lysed and the lysate was incubated with TRAP substrate buffer (50 mM sodium citrate, 50 mM citric acid, 5 mM pNPP, and 10 mM sodium tartrate; pH 4.8) at 37 °C for 20–30 min. An aliquot of the reacted solution was transferred to a 96-well plate, and the reaction was stopped with 0.1 M NaOH. TRAP activity was measured by absorbance at 405 nm using a multi-detection microplate reader (Synergy HT, BioTek Instruments).

### 2.5. Western Blotting

To determine the protein expression level, cell lysates were harvested with RIPA buffer supplemented with protease and phosphatase inhibitors. Protein concentration was quantified using the Bradford assay. A total of 7.5 µg protein for each sample was separated by 10% sodium dodecyl sulfate—polyacrylamide gel electrophoresis (SDS-PAGE) and transferred to polyvinylidene difluoride (PVDF) membranes (Bio-Rad, Hercules, CA, USA). The membranes were blocked in PBST containing 3% bovine serum albumin (BSA) for 1 h at room temperature. The membranes were then incubated overnight at 4 °C with the following primary antibodies (Santa Cruz Biotechnology, Dallas, TX, USA): BMP2/4, TGF-β, β-catenin, phospho-Smad1/5, phospho-Smad2/3, RUNX2, OSX, OPN, and β-actin for osteoblast differentiation, and RANK, phospho-NF-κB, NF-κB, CTSK, NFATc1, and MMP9 for osteoclast differentiation. After incubation, membranes were washed three times for 10 min each with PBST. The membranes were subsequently incubated with the appropriate HRP-conjugated secondary antibody (Santa Cruz Biotechnology) for 1 h at room temperature.

Protein levels were detected using a Pierce™ ECL Western Blotting Substrate (Thermo Fisher Scientific) and band images were analyzed by GE FujiFilm ImageQuant LAS-4000 Luminescent Image Analyzer (Fujifilm, Tokyo, Japan). The resulting bands were normalized using β-actin as an internal control.

### 2.6. Quantitative Real-Time PCR (qRT-PCR)

Total RNA was collected using RiboEx™ buffer (GeneAll Biotechnology, Seoul, Republic of Korea). To determine the mRNA expression level, total RNA was extracted using the HybridR^TM^ RNA isolation kit (GeneAll) according to the manufacturer’s protocol. cDNA was synthesized from total RNA using the PrimeScript 1st Strand cDNA Synthesis Kit (TaKaRa, Kusatsu, Japan). qRT-PCR analysis was performed using SYBR Green Master Mix (Thermo Fisher Scientific) in a LightCycler 480 Real-Time PCR System (Roche, Basel, Switzerland). Target mRNA expressions were normalized using β-actin. The primer sequences are shown in Supplementary Table S1.

### 2.7. Animal Experiment

All animal care and experimental procedures were approved by the Institutional Animal Care and Use Committee (IACUC) of the Korea Institute of Science and Technology (KIST-2022-153-1). Seven-week-old female C57BL/6J mice were purchased from DooYeol Biotech (Seoul, Republic of Korea) and housed at a constant temperature (25 ± 2 °C) with a 12 h light/dark cycle with food and water ad libitum. After one week of acclimation, osteoporosis was induced in the mice via bilateral ovariectomy (OVX) under isoflurane anesthesia. Sham surgery was performed under identical conditions without ovariectomy. The mice were divided randomly into five groups: Sham group (control, n = 6), OVX mice (OVX group, n = 6), OVX mice treated with 0.1 mg/kg β-estradiol and 1 mg/kg progesterone (E+P group, n = 7), OVX mice treated with 10 mg/kg QSEE (QS10 group, n = 5), and OVX mice treated with 40 mg/kg QSEE (QS40 group, n = 8). All samples were prepared in a 0.5% carboxymethyl cellulose (CMC) solution containing 0.5% sesame oil and 0.5% DMSO. Sham and OVX mice were treated with a 0.5% CMC solution containing the same additives. Two weeks after surgery, treatment was conducted 5 days a week for 12 weeks by oral administration. Body weights were measured weekly. All mice were sacrificed by cervical dislocation. Blood, uterus, and femurs were collected for further analysis. Left femurs were fixed with 10% formalin for micro-computed tomography (micro-CT), and right femurs and tibias were used for primary cell culture.

### 2.8. Primary Cell Isolation

To perform ex vivo analysis of cells from treated animals, primary bone cells were isolated. The right femur and both tibias from each mouse were dissected aseptically. For primary osteoclast precursor culture, the bone epiphyses were cut, and the marrow was collected by centrifugation. The collected bone marrow cells from each group were pooled and cultured in D-MEM (Welgene) containing FBS (Gibco) and 1% P/S (Hyclone), and 30 ng/mL M-CSF (Peprotech) to expand macrophage-like cells. Non-adherent cells were removed, and adherent cells (bone marrow-derived macrophages, BMDMs) were used as osteoclast precursors. These precursors were further differentiated with medium containing 30 ng/mL M-CSF and 50 ng/mL RANKL (Peprotech). These cells were subjected to TRAP activity and qRT-PCR to evaluate osteoclastogenic gene expression.

For primary osteoblast precursor culture, bone marrow cells were collected and the remaining bones were cut into small pieces (<1 mm^3^) in a Petri dish containing 5 mL of digestion medium (1 mg/mL collagenase II in DPBS). During a 90 min incubation, bone pieces were gently shaken every 30 min. After incubation, the digestion medium containing dissociated cells was collected and centrifuged at 1200 rpm for 5 min. The released cells and bone marrow from each group were pooled and cultured in collagen type I (Sigma-Aldrich)—coated flasks containing α-MEM (Welgene) supplemented with FBS (Gibco) and 1% P/S (Hyclone). These osteoblastic precursors were further differentiated with medium containing 10 mM β-glycerophosphate (Sigma-Aldrich) and 50 µg/mL L-ascorbic acid (Sigma-Aldrich). These cells were then analyzed for ALP activity, mineralization, Western blot, and qRT-PCR to assess osteogenic differentiation.

### 2.9. Alizarin Red Staining

Primary cells were seeded at 2 × 104 cells/well in a 24-well plate. Cells were differentiated with DM, which was replaced every 3 days for 14 days. Cells were washed with DPBS, fixed with 3.7% formalin for 2 h at room temperature, and stained with 2% Alizarin Red S (ARS) solution (pH 4.3) for 1 h in the dark. After removing ARS, the cells were dried overnight wrapped in foil. The staining area was quantified under a microscope. After quantification, the stained cells were solubilized with 10% hexadecylpyridinium chloride (Sigma-Aldrich) for 1 h at room temperature in the dark. The absorbance of the solubilized stain was measured at 504 nm using a multi-detection microplate reader (Synergy HT, BioTek Instruments).

### 2.10. Micro-Computed Tomography (Micro-CT)

All left femurs were stored in 10% formalin and scanned using SkyScan 1172 (Bruker MicroCT Corp, Kontich, Belgium). The original images were reconstructed and bone tomography was analyzed using CT analyzer software (NRecon software v1.7.3.2).

### 2.11. Histology and Immunohistochemistry (IHC) Staining

The left femurs were fixed with 10% (*v*/*v*) formalin. The samples were sectioned at 5 μm and stained with hematoxylin and eosin (H&E) and collagen type I antibody.

For H&E staining, paraffin-embedded sections were deparaffinized in xylene and rehydrated through graded alcohols. Slides were stained with Harris hematoxylin for 5 min, differentiated with 0.5% acid alcohol and blued in 0.2% ammonia water. After counterstaining with eosin Y for 2.5 min, the sections were dehydrated, cleared and mounted.

For immunohistochemical (IHC) staining, the sections were incubated with primary Collagen type I antibody (Abcam, Waltham, MA, USA) at room temperature for 3 h. Polink-2 Plus HRP Broad Spectrum DAB Detection Kit (Origene, Rockville, MD, USA) was used for specificity of IHC staining.

### 2.12. HPLC Analysis

The 70% ethanol extract of *Quercus salicina* Blume was analyzed using a high-performance liquid chromatography (HPLC) system (Agilent 1260 Infinity, Agilent Technologies, Waldbronn, Germany) equipped with a YMC-Triart C18 column (150 × 4.6 mm, particle size 5 μm, pore size 12 nm). Chromatographic separation was performed with a linear gradient elution of solvent A (0.1% acetic acid in water) and solvent B (acetonitrile) at a flow rate of 1.0 mL/min. The gradient conditions were programmed as follows: 8–10% B from 0 to 2 min, 10–30% B from 2 to 27 min, 30–90% B from 27 to 50 min, 90–100% B from 50 to 51 min, and held at 100% B from 51 to 60 min. The column temperature was maintained at 30 °C, and detection was carried out at a wavelength of 254 nm. Each sample was filtered through a 0.2 μm membrane filter before injection, and a 10 μL aliquot of the sample solution (10 mg/mL) was injected for analysis. UV absorption spectra of the major chromatographic peaks were recorded over 200~400 nm and compared with those of the reference standards. For quantitative analysis, chlorogenic acid, quercetin, and isorhamnetin were used as external reference standards and analyzed under the same HPLC conditions. Calibration curves were constructed over concentration ranges of 1–500 μg/mL for chlorogenic acid, 1–100 μg/mL for quercetin, and 1–500 μg/mL for isorhamnetin, with regression equations of y = 15469x + 26.462 (R^2^ = 0.9995), y = 20252x–46.639 (R^2^ = 0.9976), and y = 36751x + 96.781 (R^2^ = 0.9997), respectively. Peak 1 was quantified using a calibration curve of chlorogenic acid (Sigma-Aldrich, St Louis, MO, USA). Since authentic standards for the compounds corresponding to peaks 2 and 3 were unavailable, their concentrations were estimated using calibration curves of the corresponding aglycones, quercetin and isorhamnetin (Sigma-Aldrich, St Louis, MO, USA), respectively.

### 2.13. UHPLC-Q-TOF-MS Analysis and Compound Annotation

Ultra-high-performance liquid chromatography–quadrupole time-of-flight mass spectrometry (UHPLC-Q-TOF-MS) analysis was performed using an Agilent 1290 Infinity UHPLC system coupled with a Q-TOF mass spectrometer (Agilent G6550A) equipped with an electrospray ionization (ESI) source. Chromatographic separation was achieved on an Acquity UPLC HSS T3 C18 column (2.1 × 100 mm, 1.8 μm; Waters Corp., Milford, MA, USA) at the Korea Basic Science Institute (KBSI). The mobile phase consisted of solvent A (0.1% formic acid in water) and solvent B (0.1% formic acid in acetonitrile), delivered at a flow rate of 0.3 mL/min. The gradient program was as follows: 5% B from 0 to 3 min, 5–90% B from 3 to 13 min, 90% B from 13 to 15 min, re-equilibration to 5% B from 15 to 17 min, and held at 5% B until 20 min. The column temperature was maintained at 30 °C, and the injection volume was 1 μL of a 10 mg/mL sample solution. Mass spectrometric data were acquired in both positive and negative ionization modes over a mass range of *m*/*z* 100–1700 at a scan rate of 1 spectrum per second. Compound annotation was based on the combined evaluation of observed *m*/*z* values, ionization behavior in positive and negative modes, observed fragment ions, and UV absorption profiles, together with comparison with available reference standards and published spectral data. Characteristic neutral losses of 162 Da (hexose), together with aglycone-related fragment ions, were used to support glycoside annotation where applicable. Chlorogenic acid was identified by comparison with an authentic reference standard. Because authentic standards for quercetin hexoside and isorhamnetin hexoside were unavailable, peaks 2 and 3 were putatively annotated rather than definitively identified, and no specific sugar identity or glycosylation position was assigned. The principal ions used for annotation were peak 1: *m*/*z* 355.0987 in positive mode and *m*/*z* 353.0909 in negative mode; peak 2: *m*/*z* 465.0941 in positive mode and *m*/*z* 463.0908 in negative mode; and peak 3: *m*/*z* 479.1083 in positive mode and *m*/*z* 477.1044 in negative mode.

### 2.14. Statistical Analysis

All experiments were performed in triplicate or using all mice per group and repeated three times. Primary cells were cultured by mixing all mice in each group, and the experiment was repeated technologically three times. All statistical analyses in this study were performed using GraphPad Prism version 10.2.3 (GraphPad, La Jolla, CA, USA). Data are presented as mean ± standard error of the mean (SEM). Normality and homogeneity of variance were analyzed with the Shapiro–Wilk test and Brown–Forsythe test. To compare multiple groups, Welch’s ANOVA test with Dunnett’s T3 multiple comparisons test was used. The *p* < 0.05 were considered significantly different.

## 3. Results

### 3.1. QSEE Promotes Osteoblast Differentiation in MC3T3-E1 Cells

**Figure 1:**
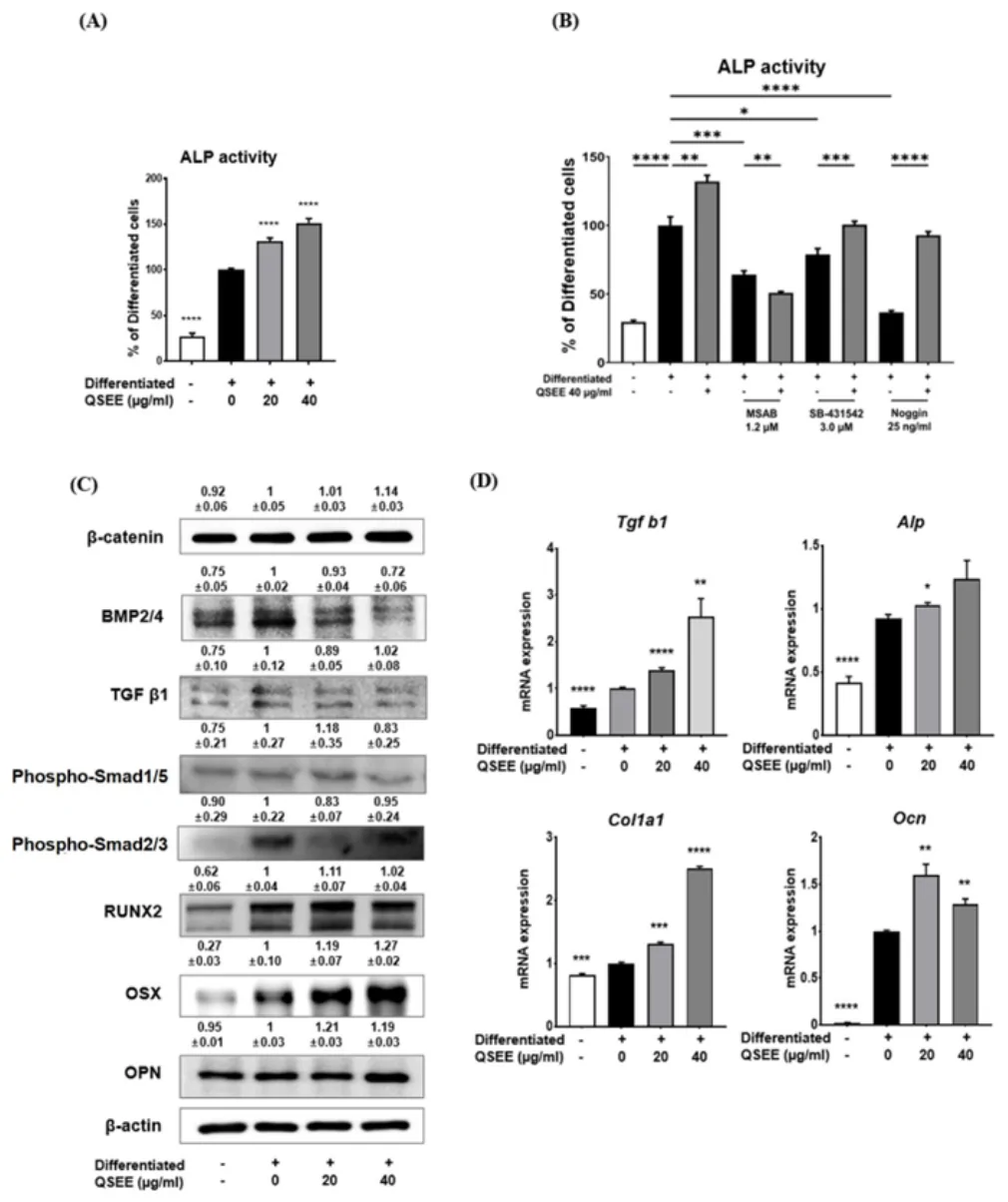
Effects of QSEE on osteoblast differentiation in MC3T3-E1 cells: (**A**) ALP activity, (**B**) inhibitor assay of TGF-β, BMP, and Wnt/β-catenin signaling, and (**C**) protein and (**D**) mRNA expression levels of osteoblastic markers as determined by Western blot and qRT-PCR. Data are presented as mean ± SEM. Compared to the differentiated control group, stars represent significant differences (* *p* < 0.05, ** *p* < 0.01, *** *p* < 0.001; and **** *p* < 0.0001).

To evaluate the effects of *Q. salicina* ethanol extract (QSEE) on osteoblast differentiation, MC3T3-E1 cells were treated with 20 µg/mL and 40 µg/mL of QSEE. These concentrations of QSEE did not alter the cell viability of MC3T3-E1 cells (Supplementary Figure S1). ALP is an important enzyme in bone formation, and high ALP activity is an indicator of osteoblast differentiation. ALP activity was significantly increased by QSEE in a dose-dependent manner (Figure 1A). Protein and mRNA levels of osteoblast differentiation markers were examined by Western blotting and qRT-PCR. QSEE treatment specifically increased Wnt/β-catenin signaling-related protein expressions such as β-catenin, RUNX2, OSX, and OPN (Figure 1C). However, TGF-β and BMP levels and their downstream targets, phospho- Smads, were not changed by QSEE treatment. qRT-PCR results showed that mRNA expression of *Tgfb1, Alp, Col1a1* and *Ocn* were increased by QSEE treatment compared to the differentiated control group (Figure 1D). However, the mRNA expression of Runx2 was not significantly changed by QSEE treatment under these conditions.

To further investigate the underlying mechanism, an inhibitor assay was performed using MSAB (Wnt inhibitor), SB431542 (TGF-β inhibitor), and Noggin (BMP inhibitor). QSEE-induced ALP activity was reduced with all three inhibitors. Among them, MSAB showed the most notable suppression, whereas SB431542 and Noggin showed only partial attenuation (Figure 1B). These results suggest that the osteogenic effect of QSEE involves Wnt/β-catenin signaling.

### 3.2. QSEE Inhibits Osteoclast Differentiation in RAW 264.7 Cells

**Figure 2:**
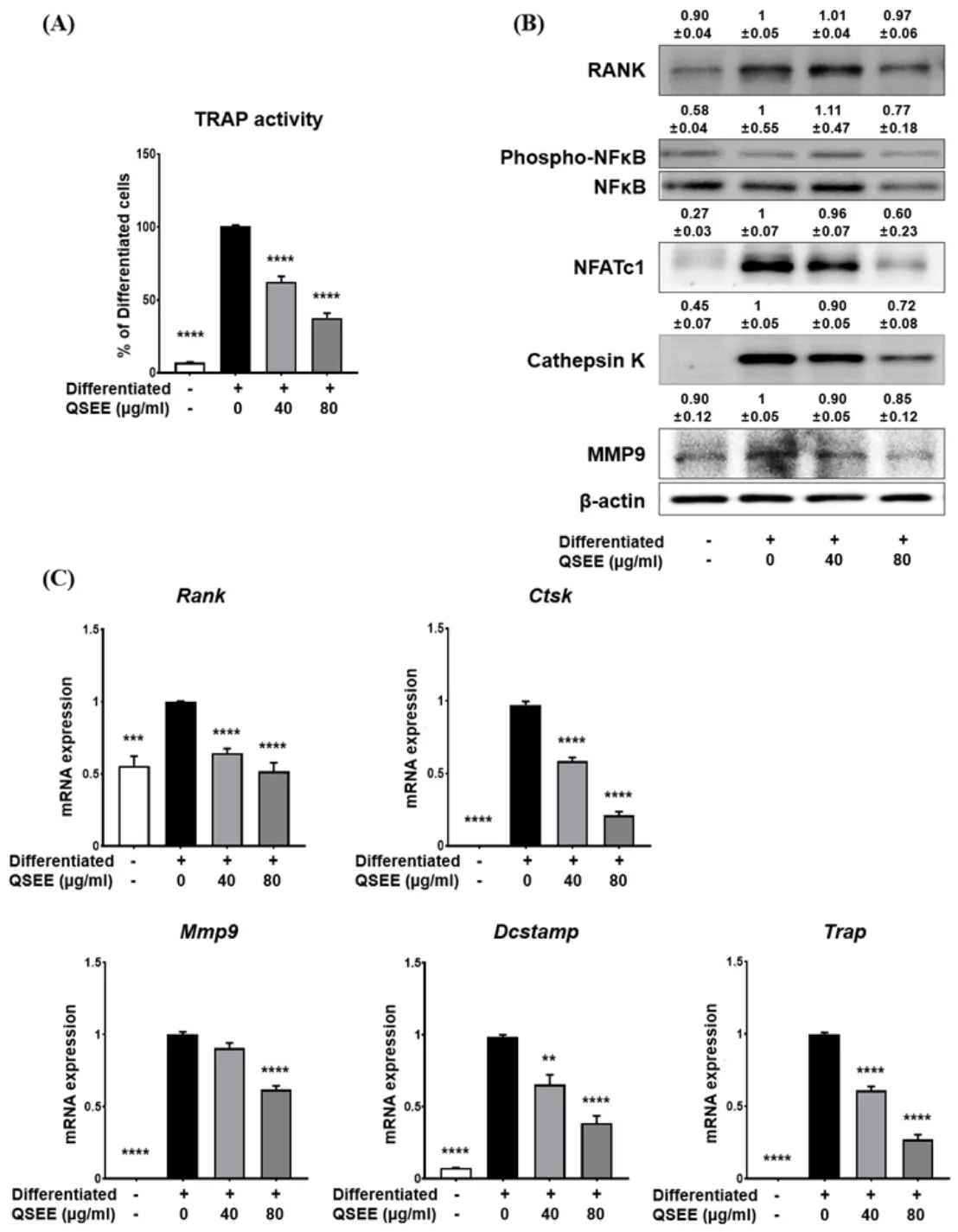
Effects of QSEE on osteoclast differentiation in RAW 264.7 cells: (**A**) TRAP activity and (**B**) protein and (**C**) mRNA expression levels of osteoclastic markers as determined by Western blot and qRT-PCR. Compared to the differentiated control group, stars represent significant differences (** *p* < 0.01, *** *p* < 0.001; and **** *p* < 0.0001).

To evaluate the effects of QSEE on RANKL-induced osteoclast differentiation, RAW 264.7 cells was treated with 40 µg/mL and 80 µg/mL of QSEE. These concentrations of QSEE did not alter the cell viability of RAW 264.7 cells (Supplementary Figure S1). TRAP is an important enzyme in bone resorption, and high TRAP activity is an indicator of osteoclast differentiation. TRAP activity was significantly decreased by QSEE in a dose-dependent manner (Figure 2A). Western blot analysis further demonstrated that QSEE treatment suppressed the protein levels of RANK, phospho-NF-κB, NFATc1, CTSK, and MMP9 (Figure 2B). Also, qRT-PCR revealed that QSEE treatment dose-dependently downregulated mRNA expression of osteoclast-related genes such as *Rank*, *Ctsk*, *Mmp9*, *DC-stamp,* and *Trap* (Figure 2C).

### 3.3. QSEE Ameliorates Ovariectomy-Induced Osteoporosis in Mice

**Figure 3:**
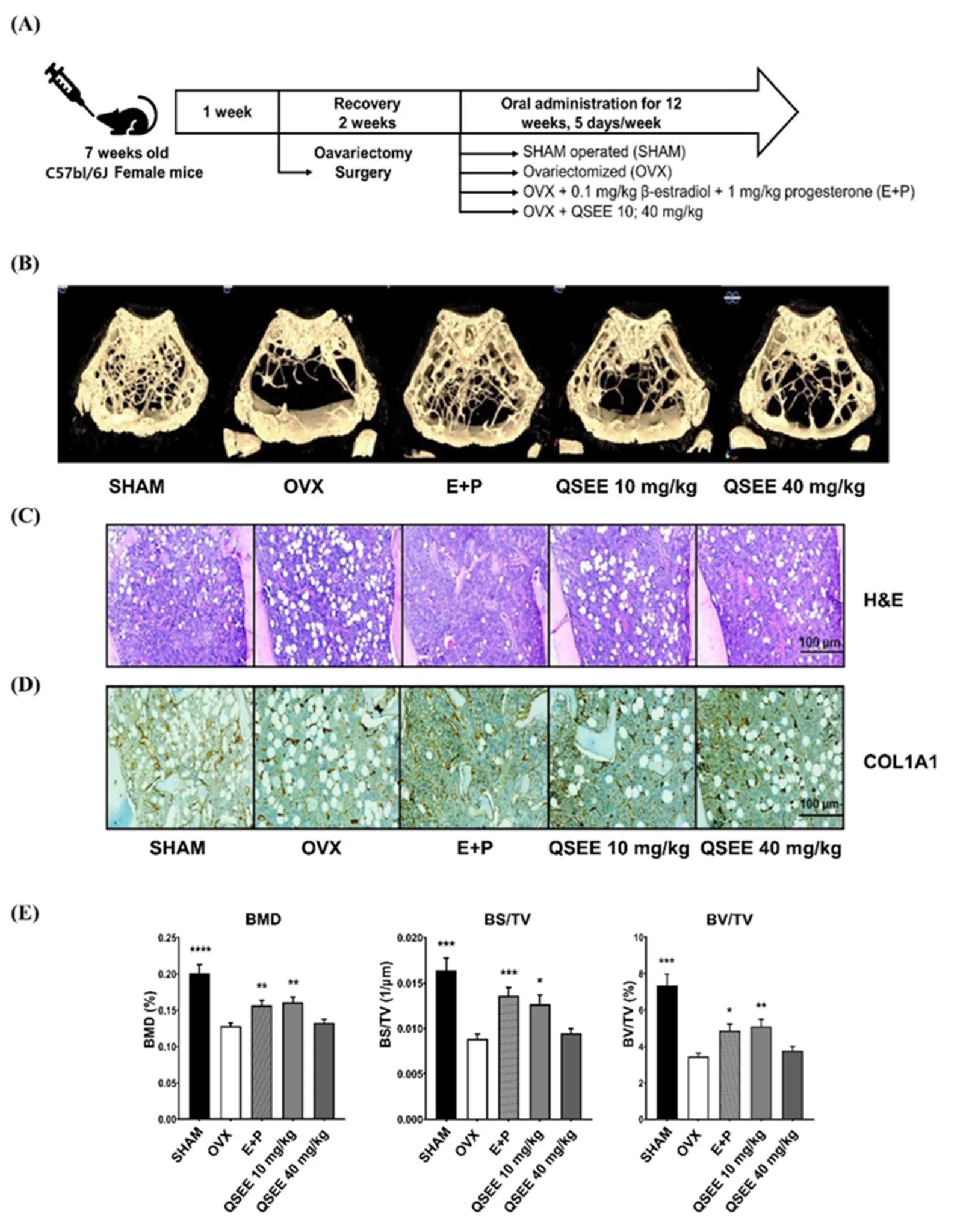
Effects of QSEE treatment on bone architecture in OVX mice. (**A**) Animal experiment design. (**B**) Micro-computed tomography images of the distal femoral region of mice. (**C**) H&E staining and (**D**) COL1A1 IHC staining. (**E**) Measurements of BMD, BS/TV, and BV/TV. Data are presented as mean ± SEM. Compared to the OVX group, stars represent significant differences (* *p* < 0.05, ** *p* < 0.01, *** *p* < 0.001; and **** *p* < 0.0001).

To determine the effect of QSEE treatment with 10, 40 mg/kg in osteoporosis, an ovariectomy (OVX) was conducted on the mice (Figure 3A). OVX reduced the weight of the uterus and E+P treatment ameliorated the decrease in the uterus, as we expected (Supplementary Figure S2A). QSEE had no impact on the weight of the uterus under OVX, indicating that the estrogenic property of QSEE is not critical. No significant change in OCN nor toxic effect in the serum was found (Supplementary Figure S2B–D). To assess bone health, micro-CT analysis of the left femur was performed (Figure 3B). QSEE administration of 10 mg/kg recovered bone parameters decreased by OVX, including BMD, BS/TV, BV/TV, bone volume, bone surface, intersection surface and trabecular number, while 40 mg/kg of QSEE did not alter them. (Figure 3D, Supplementary Figure S3). 

To evaluate femoral status, mouse femur sections were stained with H&E and COL1A1. H&E staining indicates the restoration of the trabecular area. COL1A1 is a protein involved in bone formation from early to late stages, and its staining intensity indicates osteogenic activity. H&E staining showed a decrease in trabecular area by OVX, which was restored by QSEE treatment (Figure 3C). The level of COL1A1-positive cells was decreased by OVX but increased in the QSEE-treated group (Figure 3C).

### 3.4. QSEE Regulates Osteoblast and Osteoclast Differentiation in Primary Cells

**Figure 4:**
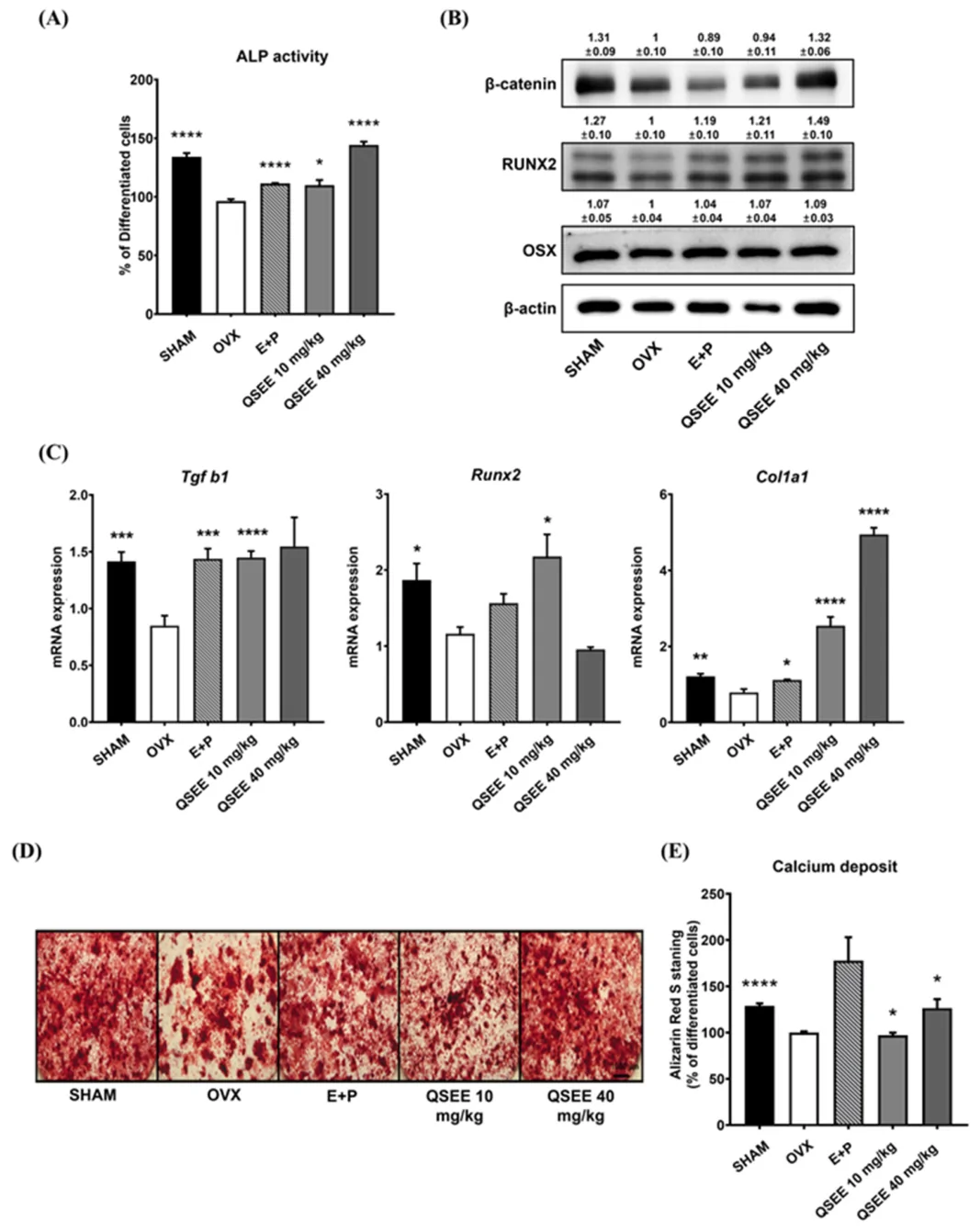
Effects of QSEE on osteoblast differentiation in primary cells: (**A**) ALP activity and (**B**) protein and (**C**) mRNA expression levels of osteoblastic markers as determined by Western blot and RT-PCR. (**D**) Estimation of mineralization (calcium deposit) quantification and (**E**) intensity of Alizarin red S staining. Compared to the OVX group, stars represent significant differences (* *p* < 0.05, ** *p* < 0.01, *** *p* < 0.001, and **** *p* < 0.0001).

Osteoblast differentiation was assessed in primary cells isolated from OVX mice to evaluate the osteogenic effects of QSEE. ALP activity was significantly increased in the QSEE-treated group compared to the OVX group, in a dose-dependent manner (Figure 4A). The protein levels of the osteoblastic markers β-catenin, RUNX2, and OSX were decreased by OVX and increased by 40 mg/kg QSEE treatment in primary cells (Figure 4B). The mRNA expression levels of the major osteoblastic markers *Tgfb1*, *Runx2*, and *Col1a1* were significantly increased by 10 mg/kg QSEE treatment in primary cells (Figure 4C). Through ARS staining, calcium deposits were shown to be decreased by OVX and increased in the 40 mg/kg QSEE-treated group (Figure 4D,E).

**Figure 5:**
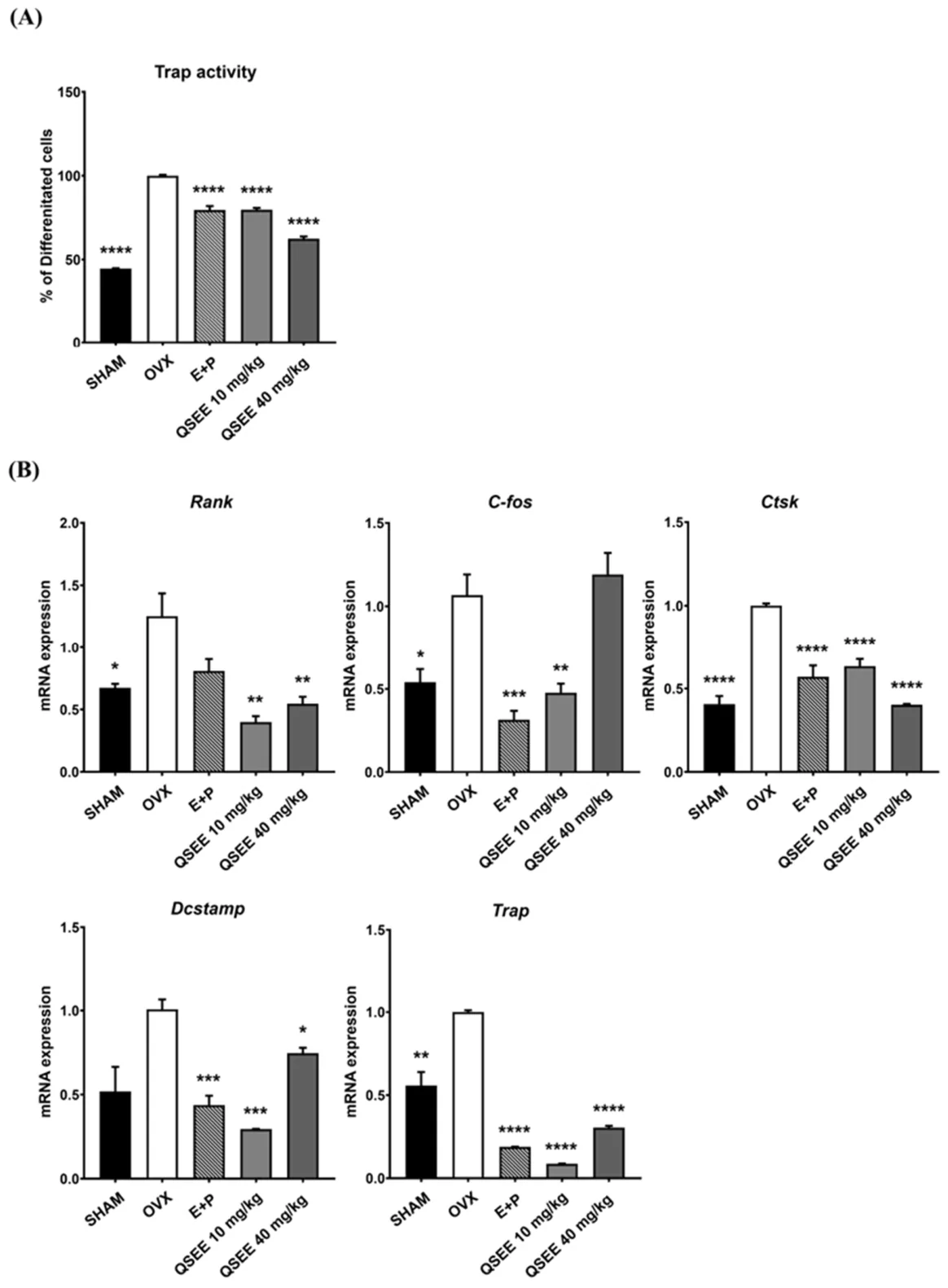
Effects of QSEE on osteoclast differentiation in primary cells: (**A**) Trap activity and (**B**) mRNA expression levels of osteoclastic markers as determined by RT-PCR. Compared to the OVX group, stars represent significant differences (* *p* < 0.05, ** *p* < 0.01, *** *p* < 0.001, and **** *p* < 0.0001).

To assess the inhibitory effect of QSEE on osteoclastogenesis, osteoclast differentiation was induced in primary cells isolated from mice treated with QSEE. TRAP activity was significantly decreased in the QSEE-treated group compared to the OVX group, in a dose-dependent manner (Figure 5A). The mRNA expression levels of major osteoclastic markers, *Rank*, *c-fos*, *Ctsk*, *DC-stamp,* and *Trap* were decreased in the 10 mg/kg QSEE-treated group, while *Rank*, *Ctsk*, *DC-stamp,* and *Trap* were altered in the 40 mg/kg QSEE group (Figure 5B).

To further investigate whether the inhibitory effects of QSEE on osteoclastogenesis were mediated by osteoblast-derived regulatory factors, ex vivo analysis was conducted to confirm OPG and RANKL expression. Western blot analysis showed that there was no significant difference in the OPG/RANKL ratio between the Sham and OVX groups. While QSEE treatment slightly increased the OPG/RANKL ratio in a dose-dependent manner, the change was not statistically significant (Supplementary Figure S4). These findings suggest that the inhibitory effects of QSEE on osteoclast differentiation are not mediated by modulation of osteoblast-derived OPG/RANKL signaling.

### 3.5. Phytochemical Profile and Quantification of QSEE

**Figure 6:**
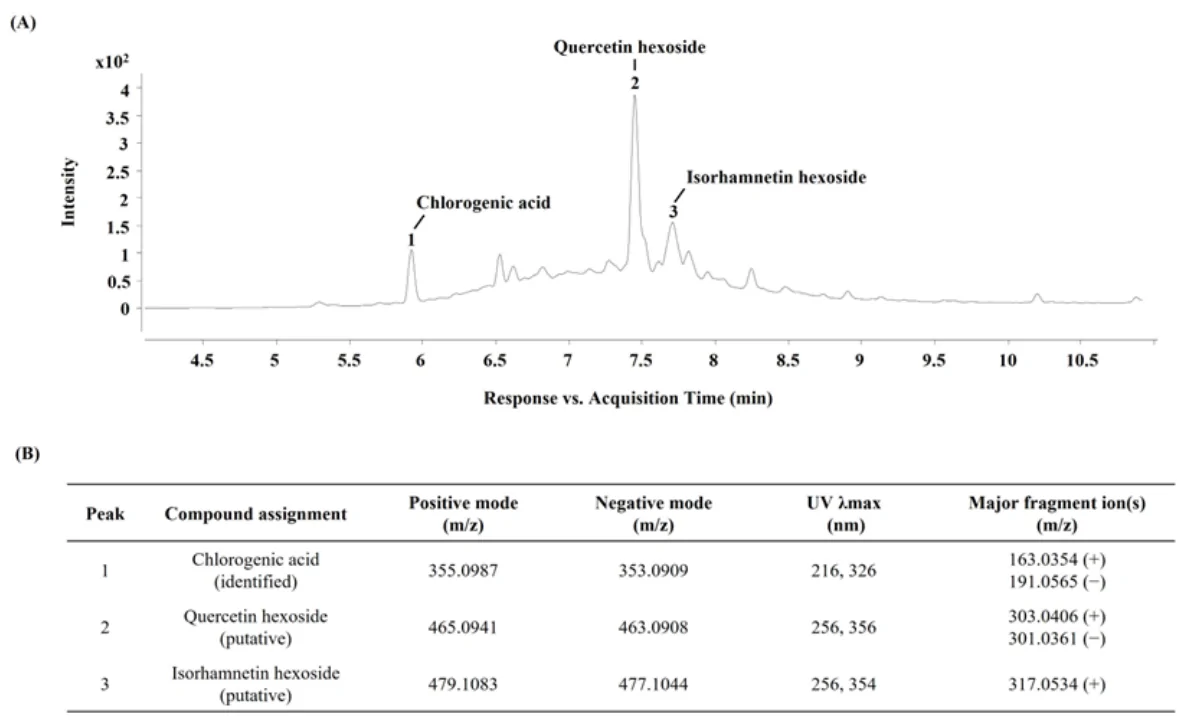
UHPLC–Q-TOF–MS chromatogram of the QSEE. (**A**) Representative chromatogram showing three major peaks. (**B**) Summary of major compounds in QSEE using positive and negative ionization modes.

The chemical constituents of the *Quercus salicina* 70% ethanol extract were analyzed by UHPLC–Q-TOF–MS in both positive and negative ionization modes, and the UV spectral profiles of the corresponding HPLC peaks were evaluated to support compound annotation (Figure 6). Three major peaks were observed. Peak 1 was detected at *m/z* 355.0987 in positive mode and *m/z* 353.0909 in negative mode. Its UV absorption maxima were observed at 216 and 326 nm, and the UV spectral profile and HPLC retention behavior were consistent with those of the chlorogenic acid reference standard. In addition, fragment ions at *m/z* 163.0354 in positive mode and *m/z* 191.0565 in negative mode were consistent with the reported fragmentation behavior of chlorogenic acid. Thus, peak 1 was identified as chlorogenic acid. Peak 2 exhibited ions at *m/z* 465.0941 in positive mode and *m/z* 463.0908 in negative mode. The UV absorption profile of peak 2 showed maxima at 256 and 356 nm and was consistent with that of a flavonol-type compound. Characteristic fragment ions at *m/z* 303.0406 in positive mode and *m/z* 301.0361 in negative mode, corresponding to the quercetin aglycone, were observed following a neutral loss of 162 Da from the precursor ions. Based on the combined *m/z*, UV, and fragmentation data, peak 2 was putatively annotated as quercetin hexoside. Peak 3 appeared at *m/z* 479.1083 in positive mode and *m/z* 477.1044 in negative mode. Its UV absorption maxima at 256 and 354 nm showed a spectral profile consistent with that of a flavonol-type compound, and a fragment ion at *m/z* 317.0534 attributable to the isorhamnetin aglycone was observed in positive ionization following a neutral loss of 162 Da, consistent with a hexose moiety. Peak 3 was therefore putatively annotated as isorhamnetin hexoside. Because authentic standards of the two glycosylated compounds were not available, the assignments of peaks 2 and 3 should be regarded as putative annotations and do not establish the exact sugar identity or glycosylation position.

Quantitative HPLC analysis showed that chlorogenic acid (peak 1) was present at 4.2433 ± 0.2854 mg/g dry extract. Peak 2, putatively annotated as quercetin hexoside, was estimated at 15.2900 ± 0.7923 mg/g dry extract using the quercetin calibration curve, whereas peak 3, putatively annotated as isorhamnetin hexoside, was estimated at 1.3567 ± 0.1528 mg/g dry extract using the isorhamnetin calibration curve (Supplementary Figure S5). These values provide quantitative marker levels for chemical characterization of QSEE; however, the values for peaks 2 and 3 represent aglycone equivalents rather than absolute concentrations determined using authentic glycoside standards. Taken together, the chromatographic, UV spectral, and mass spectrometric data support chlorogenic acid and the putatively annotated quercetin hexoside and isorhamnetin hexoside as major phenolic constituents of QSEE [15].

## 4. Discussion

Despite the increasing risks of osteoporosis with an increasingly aging population, current clinical treatments, such as parathyroid hormone analogs and bisphosphonates, primarily target either bone formation or bone resorption, and despite their efficacy, they present limitations associated with long-term administration [3]. In addition, current studies have started to focus on the imbalance between osteoblast and osteoclast activity as a key pathogenic mechanism underlying osteoporosis [19]. Here, our study demonstrates that QSEE modulates both osteoblast and osteoclast differentiation. QSEE enhanced osteoblast differentiation by increasing osteogenic markers such as RUNX2 and ALP, while simultaneously suppressing osteoclast differentiation through the downregulation of NFATc1 and TRAP. These dual effects were reflected in the OVX mouse model, in which QSEE administration improved bone microarchitecture by modulating the population of osteoblasts and osteoclasts within bone tissue. In this context, our findings highlight the potential value of *Quercus salicina* Blume ethanol extract (QSEE) as a phytotherapeutic candidate capable of exerting dual regulatory effects on bone metabolism.

Osteoblasts play a pivotal role in maintaining bone density. Three major signaling pathways regulate osteoblast differentiation: Wnt/β-catenin, TGF-β, and BMP pathways [6,7,20]. Activation of these pathways upregulates RUNX2 and OSX, key transcription factors for osteoblast differentiation [10,21]. Also, RUNX2 and OSX upregulate the expression of osteogenic markers such as ALP, COL1A1, and OCN [9]. ALP and COL1A1 are early markers associated with matrix maturation and mineralization during the initial stages of osteoblast development [22,23]. Our results showed that QSEE treatment elevated the protein levels of RUNX2 and its downstream target genes, including OSX and OPN, thereby promoting osteoblast differentiation in vitro. Mechanistic inhibitor studies suggest that the Wnt/β-catenin signaling pathway is the critical driver of these effects. QSEE treatment increased cellular β-catenin levels, and MSAB, a specific inhibitor of the Wnt/β-catenin pathway, substantially suppressed QSEE-induced ALP activity. Interestingly, while the Wnt pathway appears to be a major driver, the TGF-β and BMP pathways may also be involved to a lesser extent. Our findings that inhibitors of TGF-β (SB431542) and BMP (Noggin) also partially, but significantly, attenuated QSEE-induced ALP activity, along with increased Tgf b1 mRNA expression, provide additional evidence for the involvement of TGF-β and BMP pathways on QSEE effect. These findings indicate that QSEE promotes osteoblast differentiation through substantial involvement of all three signaling pathways: Wnt/β-catenin, TGF-β and BMP pathways.

Because bone homeostasis also depends on bone resorption, the regulation of osteoclasts is critical for maintaining bone density. The binding of RANKL to RANK triggers osteoclast differentiation and induces the expression of the key transcription factor NFATc1 on osteoclast differentiation [24]. NFATc1 is a master regulator of osteoclast-specific marker expression such as RANK, CTSK, MMP9, DC-stamp, and TRAP [25]. CTSK and MMP9 are essential for bone matrix degradation and osteoclast migration, while DC-stamp mediates precursor cell fusion [26]. QSEE treatment significantly reduced the expression of NFATc1 and its downstream genes, including RANK, CTSK, MMP9, DC-stamp, and TRAP both in vitro and ex vivo. Consequently, QSEE resulted in a decrease in TRAP activity in vitro, and reduced osteoclast differentiation and population in vivo. These findings suggest that QSEE suppresses osteoclast differentiation through modulation of NFATc1 activation and its downstream transcriptional network.

Osteoclast differentiation is regulated both directly and through osteoblast-mediated mechanisms [27]. Osteoblasts modulate osteoclastogenesis through direct cell-to-cell contact or by secreting regulatory factors such as RANKL and OPG [28]. RANKL binds to RANK on osteoclast precursors to promote osteoclast differentiation, while OPG acts as a decoy receptor inhibiting this interaction [29]. Therefore, osteoblasts can regulate the production and differentiation of osteoclasts through the OPG/RANKL system. However, QSEE treatment did not significantly alter the OPG/RANKL ratio but only reduced RANKL expression ex vivo. In addition, QSEE suppressed osteoclast differentiation in vitro, in which only osteoclast precursor cells are cultured. These data suggest that the anti-osteoclastogenic effects of QSEE are primarily due to a direct inhibitory action on osteoclast precursor cells, but are not mediated by osteoblasts.

Interestingly, in the OVX model, the lower dose of QSEE (10 mg/kg) appeared to yield comparable or slightly greater improvements in certain bone microarchitecture parameters than the higher dose (40 mg/kg). Several factors may explain this non-linear response in vivo. First, high concentrations in vitro showed slight reductions in cell viability or marker activity thresholds, suggesting that excessively high systemic doses might approach limits of optimal tolerability. Second, 10 mg/kg QSEE is the saturated concentration near a plateau or saturation level in mice. Such a non-linear or biphasic dose–response has been reported for polyphenol-rich botanical extracts, where beneficial effects at lower doses may be attenuated at higher doses due to factors such as limited oral absorption, saturation of exposure, changes in gut microbial metabolism, or matrix effects related to tannins that can alter bioavailability and nutrient interactions [30]. In addition, complex extracts may contain constituents with counterbalancing activities that become more prominent at higher doses [31,32]. Collectively, these considerations suggest the presence of an optimal therapeutic window for QSEE, underscoring the need for follow-up studies to refine dose–response relationships and to integrate pharmacokinetic/pharmacodynamic (PK/PD) assessments.

Three predominant phenolic constituents from QSEE were identified: chlorogenic acid, quercetin hexoside, and isorhamnetin hexoside. Previous studies have reported that chlorogenic acid, quercetin and isorhamnetin exert anti-osteoporotic properties by regulating bone remodeling [33,34,35,36]. Thus, the presence of these compounds provides a plausible chemical foundation for the dual regulatory effects of QSEE on bone metabolism. Although UHPLC–Q-TOF–MS analysis allowed us to identify the major compound peaks of QSEE, the specific contribution of each compound to the observed osteogenic and anti-osteoclastogenic activities remains unclear. Future studies employing bioassay guided fractionation and purified compounds will be necessary to determine the individual bioactive components and their precise mechanisms of action.

This study has several limitations. First, the specific active molecules in QSEE were not fully isolated and characterized, meaning that individual biological activities could not be definitively assigned to single compounds. Second, the use of pooled primary cells, necessitated by the limited yield of cells obtainable from individual mice, restricts mouse-to-mouse variability tracking. Furthermore, the mild cytotoxicity observed in vitro at higher concentrations did not translate into overt systemic toxicity in vivo, indicating that safety profiles require more rigorous evaluation. Therefore, further studies utilizing bioactivity-guided fractionation and pharmacokinetic tracking are required to clarify the precise mechanisms of action of QSEE.

## 5. Conclusions

In conclusion, this study demonstrates that QSEE exerts a dual regulatory effect on bone remodeling by promoting osteogenesis via RUNX2 and inhibiting osteoclastogenesis through suppression of NFATc1. QSEE treatment significantly improved bone microarchitecture in OVX mice, and its molecular effects were further validated through in vitro and ex vivo studies. These findings provide strong evidence supporting the potential of QSEE as a natural product-based candidate to develop anti-osteoporosis agents. However, further studies, including isolation of active compounds and clinical investigations, will be essential to fully establish its therapeutic value.

## Data Availability

The original contributions presented in this study are included in the article/Supplementary Materials. Further inquiries can be directed to the corresponding authors.

## Supplementary Materials

The following supporting information can be downloaded at https://www.mdpi.com/article/10.3390/nu18213578/s1. Figure S1: Cell viability assay; Figure S2: Effects of QSEE treatment on physiological markers in ovariectomized mice; Figure S3: Effects of QSEE treatment on bone parameters in ovariectomized mice; Figure S4: OPG/RANKL ratio in primary cells; Figure S5: UV absorption spectra of the three major peaks detected in the HPLC chromatogram of QSEE; Table S1: Primer sequences for qRT-PCR.

## Abbreviations

The following abbreviations are used in this manuscript:

ALP	Alkaline Phosphatase
BMP	Bone Morphogenetic Protein
BMD	Bone Mineral Density
BV/TV	Bone Volume/Total Volume
BS/TV	Bone Surface/Total Volume
*C*-fos	Proto-oncogene c-Fos
CMC	Carboxymethyl Cellulose
Col1a1	Collagen Type I Alpha 1
CTSK	Cathepsin K
DC-STAMP	Dendritic Cell-Specific Transmembrane Protein
H&E	Hematoxylin and Eosin
IHC	Immunohistochemistry
LRP5/6	Low-Density Lipoprotein Receptor-Related Protein 5/6
MAPKs	Mitogen-Activated Protein Kinases
M-CSF	Macrophage Colony-Stimulating Factor
Micro-CT	Micro-Computed Tomography
MMP9	Matrix Metalloproteinase 9
OCN	Osteocalcin
OPG	Osteoprotegerin
OPN	Osteopontin
OSCAR	Osteoclast-associated Receptor
OSX	Osterix
OVX	Ovariectomized
QSEE	*Quercus salicina* Ethanol Extract
RANK	Receptor Activator of Nuclear Factor Kappa-B
RANKL	Receptor Activator of Nuclear Factor Kappa-B Ligand
RUNX2	Runt-Related Transcription Factor 2
TGF-β	Transforming Growth Factor Beta
Tb.Th	Trabecular Thickness
Tb.N	Trabecular Number
Tb.Sp	Trabecular Spacing
TRAF	TNF Receptor-Associated Factor
TRAP	Tartrate-Resistant Acid Phosphatase

## References

[1] Atik O.S., Gunal I., Korkusuz F. (2006). Burden of Osteoporosis. Clin. Orthop. Relat. Res..

[2] Kim J.-M., Lin C., Stavre Z., Greenblatt M.B., Shim J.-H. (2020). Osteoblast-Osteoclast Communication and Bone Homeostasis. Cells.

[3] Skjødt M.K., Frost M., Abrahamsen B. (2019). Side effects of drugs for osteoporosis and metastatic bone disease. Br. J. Clin. Pharmacol..

[4] An J., Yang H., Zhang Q., Liu C., Zhao J., Zhang L., Chen B. (2016). Natural products for treatment of osteoporosis: The effects and mechanisms on promoting osteoblast-mediated bone formation. Life Sci..

[5] Qin Y., Guan J., Zhang C. (2014). Mesenchymal stem cells: Mechanisms and role in bone regeneration. Postgrad. Med. J..

[6] Wu M., Wu S., Chen W., Li Y.-P. (2024). The roles and regulatory mechanisms of TGF-β and BMP signaling in bone and cartilage development, homeostasis and disease. Cell Res..

[7] Nishita M., Hashimoto M.K., Ogata S., Laurent M.N., Ueno N., Shibuya H., Cho K.W.Y. (2000). Interaction between Wnt and TGF-β signalling pathways during formation of Spemann’s organizer. Nature.

[8] Song D., He G., Shi Y., Ni J., Long F. (2021). Functional interaction between Wnt and Bmp signaling in periosteal bone growth. Sci. Rep..

[9] Komori T. (2019). Regulation of Proliferation, Differentiation and Functions of Osteoblasts by Runx2. Int. J. Mol. Sci..

[10] Pérez-Campo F.M., Santurtún A., García-Ibarbia C., Pascual M.A., Valero C., Garcés C., Sañudo C., Zarrabeitia M.T., Riancho J.A. (2016). Osterix and RUNX2 are Transcriptional Regulators of Sclerostin in Human Bone. Calcif. Tissue Int..

[11] Boyle W.J., Simonet W.S., Lacey D.L. (2003). Osteoclast differentiation and activation. Nature.

[12] Kim J.H., Kim N. (2016). Signaling Pathways in Osteoclast Differentiation. Chonnam Med. J..

[13] Teitelbaum S.L., Ross F.P. (2003). Genetic regulation of osteoclast development and function. Nat. Rev. Genet..

[14] Takayanagi H., Kim S., Koga T., Nishina H., Isshiki M., Yoshida H., Saiura A., Isobe M., Yokochi T., Inoue J.-I. (2002). Induction and Activation of the Transcription Factor NFATc1 (NFAT2) Integrate RANKL Signaling in Terminal Differentiation of Osteoclasts. Dev. Cell.

[15] Aung T., Bibat M.A.D., Zhao C.-C., Eun J.-B. (2020). Bioactive compounds and antioxidant activities of *Quercus salicina* Blume extract. Food Sci. Biotechnol..

[16] Wang G., Yin S., Ding Y., Lin Z., Li Y. (2022). *Quercus salicina* Blume: Research Progress in Chemistry and Pharmacodynamics (1959–2021). Chem. Biodivers..

[17] Kim J.-I., Kim H.-H., Kim S., Lee K.-T., Ham I.-H., Whang W.-K. (2008). Antioxidative compounds from *Quercus salicina* Blume Stem. Arch. Pharmacal Res..

[18] Moriyama M.T., Miyazawa K., Noda K., Oka M., Tanaka M., Suzuki K. (2007). Reduction in oxalate-induced renal tubular epithelial cell injury by an extract from *Quercus salicina* Blume/*Quercus stenophylla* Makino. Urol. Res..

[19] Appelman-Dijkstra N.M., Papapoulos S.E. (2015). Modulating Bone Resorption and Bone Formation in Opposite Directions in the Treatment of Postmenopausal Osteoporosis. Drugs.

[20] Kim J.H., Liu X., Wang J., Chen X., Zhang H., Kim S.H., Cui J., Li R., Zhang W., Kong Y. (2013). Wnt signaling in bone formation and its therapeutic potential for bone diseases. Ther. Adv. Musculoskelet. Dis..

[21] AlMuraikhi N., Binhamdan S., Alaskar H., Alotaibi A., Tareen S., Muthurangan M., Alfayez M. (2023). Inhibition of GSK-3β Enhances Osteoblast Differentiation of Human Mesenchymal Stem Cells through Wnt Signalling Overexpressing Runx2. Int. J. Mol. Sci..

[22] Liu W., Zhang L., Xuan K., Hu C., Li L., Zhang Y., Jin F., Jin Y. (2018). Alkaline Phosphatase Controls Lineage Switching of Mesenchymal Stem Cells by Regulating the LRP6/GSK3β Complex in Hypophosphatasia. Theranostics.

[23] Kannan S., Ghosh J., Dhara S.K. (2020). Osteogenic differentiation potential of porcine bone marrow mesenchymal stem cell subpopulations selected in different basal media. Biol. Open.

[24] Wada T., Nakashima T., Hiroshi N., Penninger J.M. (2006). RANKL–RANK signaling in osteoclastogenesis and bone disease. Trends Mol. Med..

[25] Kim J.H., Kim N. (2014). Regulation of NFATc1 in Osteoclast Differentiation. J. Bone Metab..

[26] Zhu G., Chen W., Tang C.Y., McVicar A., Edwards D., Wang J., McConnell M., Yang S., Li Y., Chang Z. (2022). Knockout and Double Knockout of Cathepsin K and Mmp9 reveals a novel function of Cathepsin K as a regulator of osteoclast gene expression and bone homeostasis. Int. J. Biol. Sci..

[27] Zhu S., Chen W., Masson A., Li Y.-P. (2024). Cell signaling and transcriptional regulation of osteoblast lineage commitment, differentiation, bone formation, and homeostasis. Cell Discov..

[28] Chen X., Wang Z., Duan N., Zhu G., Schwarz E.M., Xie C. (2018). Osteoblast-osteoclast interactions. Connect. Tissue Res..

[29] Peng S., Liu X.S., Huang S., Li Z., Pan H., Zhen W., Luk K.D., Guo X.E., Lu W.W. (2011). The cross-talk between osteoclasts and osteoblasts in response to strontium treatment: Involvement of osteoprotegerin. Bone.

[30] Perrone P., D’Angelo S. (2025). Hormesis and health: Molecular mechanisms and the key role of polyphenols. Food Chem. Adv..

[31] Meletiadis J., Stergiopoulou T., O’Shaughnessy Elizabeth M., Peter J., Walsh Thomas J. (2007). Concentration-Dependent Synergy and Antagonism within a Triple Antifungal Drug Combination against Aspergillus Species: Analysis by a New Response Surface Model. Antimicrob. Agents Chemother..

[32] Toledo E., Félix C., Vicente T.F.L., Augusto A., Félix R., Toledo B., Silva J., Trindade C., Raimundo D., Lemos M.F.L. (2023). Seaweed Extracts to Control Postharvest Phytopathogenic Fungi in Rocha Pear. J. Fungi.

[33] Zhou R.P., Lin S.J., Wan W.B., Zuo H.L., Yao F.F., Ruan H.B., Xu J., Song W., Zhou Y.C., Wen S.Y. (2016). Chlorogenic Acid Prevents Osteoporosis by Shp2/PI3K/Akt Pathway in Ovariectomized Rats. PLoS ONE.

[34] Kwak S.C., Lee C., Kim J.Y., Oh H.M., So H.S., Lee M.S., Rho M.C., Oh J. (2013). Chlorogenic acid inhibits osteoclast differentiation and bone resorption by down-regulation of receptor activator of nuclear factor kappa-B ligand-induced nuclear factor of activated T cells c1 expression. Biol. Pharm. Bull..

[35] Wong S.K., Chin K.Y., Ima-Nirwana S. (2020). Quercetin as an Agent for Protecting the Bone: A Review of the Current Evidence. Int. J. Mol. Sci..

[36] Jiao H., Zheng K. (2025). Isorhamnetin ameliorates ovariectomy-induced osteoporosis in female rats by regulating the receptor activator of nuclear factor kappa B ligand, osteoprotegerin, bone morphogenetic protein 2 and runt-related transcription factor 2 signaling pathway. J. Vet. Med. Sci..

