# Supplementary material for: Quercus salicina Blume Inhibits Bone Loss via Dual Effects on Osteoblasts and Osteoclasts
Source: Nutrients. 2026 Oct 29;18(21):3578. doi: 10.3390/nu18213578 (PMC13764933; doi:10.3390/nu18213578)
Supplement: Supplementary file 1 [file nutrients-18-03578-s001.zip › nutrients-4376062-supplementary.pdf]

Supplementary Table S1. Primer sequences for qRT-PCR

Genes	Direction	Primer
Tgfb1	Reverse	TGG AGC AAC ATG TGG AAC TC
Tgfb1	Forward	GTC AGC AGC CGG TTA CCA
Runx2	Reverse	TCC ACA AGG ACA GAG TCA GAT TAC
Runx2	Forward	TGG CTC AGA TAG GAG GGG TA
Alp	Reverse	GAT CAT TCC CAC GTT TTC AC
Alp	Forward	TGC GGG CTT GTG GGA CCT GC
Col1a1	Reverse	GAC GCC ATC AAG GTC TAC TG
Col1a1	Forward	ACG GGA ATC CAT CGG TCA
Ocn	Reverse	AGA CTC CGG CGC TAC CTT
Ocn	Forward	CTC GTC ACA AGC AGG GTT AAG
Rank	Reverse	TCG TCC ACA GAC AAA TGC AAA
Rank	Forward	GTG TGC TTC TAG CTT TCC AAG G
C-fos	Reverse	TAC TAC CAT TCC CCA GCC GA
C-fos	Forward	GCT GTC ACC GTG GGG ATA AA
Ctsk	Reverse	CAC CCA GTG GGA GCT ATG GAA
Ctsk	Forward	GCC TCC AGG TTA TGG GCA GA
Mmp9	Reverse	GAA GGC AAA CCC TGT GTG TT
Mmp9	Forward	AGA GTA CTG CTT GCC CAG GA
Dcstamp	Reverse	GGA ACC TAA GCG GAA CTT AGA CA
Dcstamp	Forward	GCT AGG GCT TCG TGG AAA CA
Trap	Reverse	ACC TTG GCA ACG TCT CTG CAC
Trap	Forward	GTC CAG CAT AAA GAT GGC CAC A
β-actin	Reverse	TTC TTT GCA GCT CCT TCG TTG CCG
β-actin	Forward	TGG ATG GCT ACG TAC ATG GCT GGG

Supplementary Materials and methods

S. 2.1. Cell viability

MC3T3-E1 cells and RAW 264.7 cells were seeded at 1×10^4 cells and 5×10^3 cells per well in 96-well plates, respectively. After 24 h of incubation, the cells were pretreated with QSEE (0–200 μg/mL) for 72 h. Cell viability was assessed using the EZ-Cytox assay (DoGenBio, Seoul, Korea) according to the manufacturer's instructions. Briefly, 10 μL of EZ-Cytox solution was added to each well, followed by incubation for 1 h at 37 °C in a 5% CO_2 atmosphere. Absorbance was measured at 450 nm using a Varioskan LUX microplate reader (Thermo Fisher Scientific).

S. 2.2. Serum parameters

ALT, AST, and OCN activity were measured by Alanine Aminotransferase (ALT/GPT), Aspartate Aminotransferase (AST/GOT) Activity Assay Kits (Elabscience Biotechnology Co., Ltd., Wuhan, China), and Gla-type osteocalcin (Gla-OC) EIA Kit (TaKaRa) accordingly to manufacturer instructions.

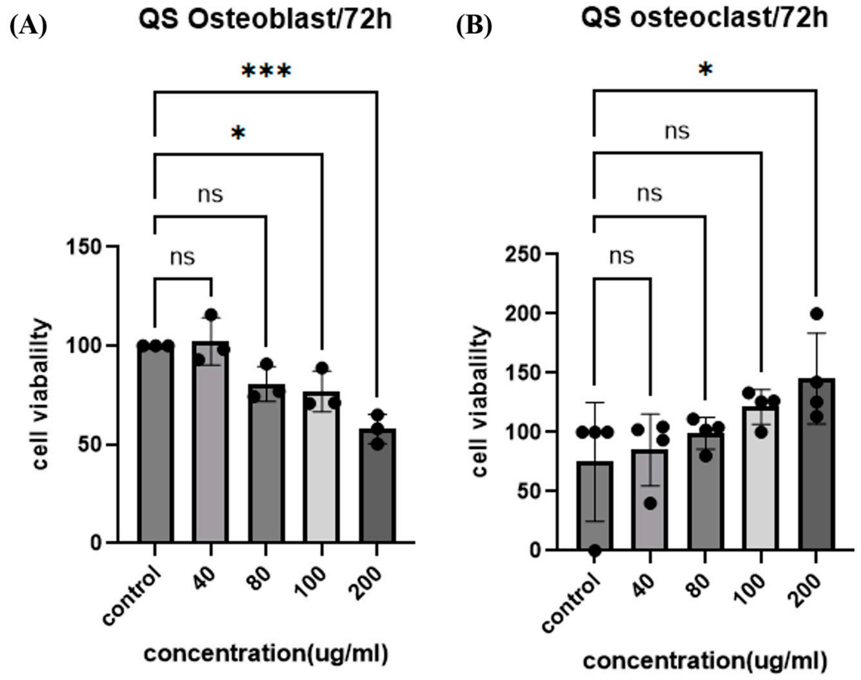

Supplementary Figure S1. **Cell viability assay** in (A) osteoblast and (B) osteoclasts

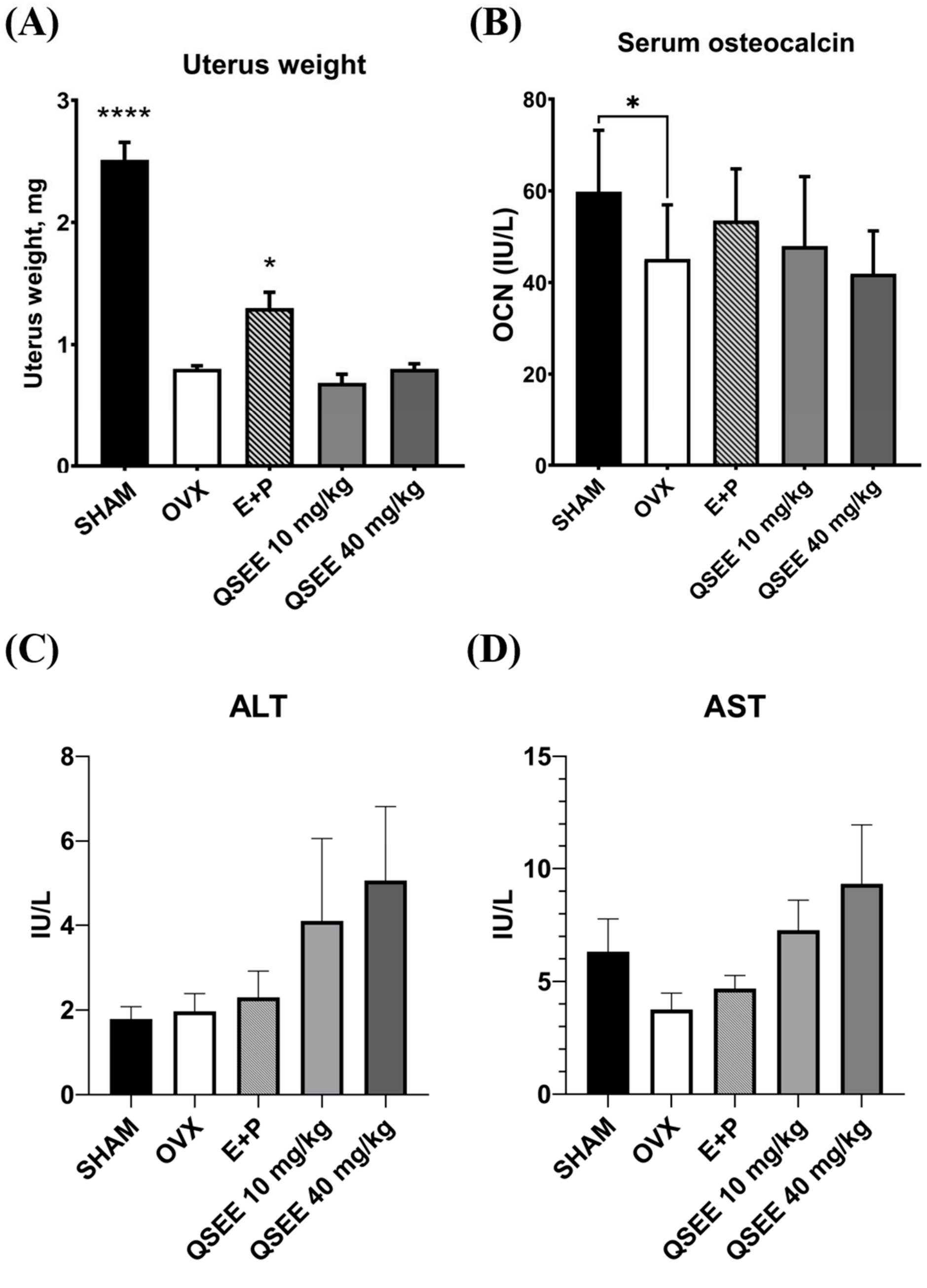

Supplementary Figure S2. Effects of QSEE treatment on physiological markers in ovariectomized mice (A) Uterus weight. (B) OCN. (C) ALT and (D) AST level in the serum. Compared to the OVX group, stars represent significant differences (* $p < 0.05$, **** $p < 0.0001$).

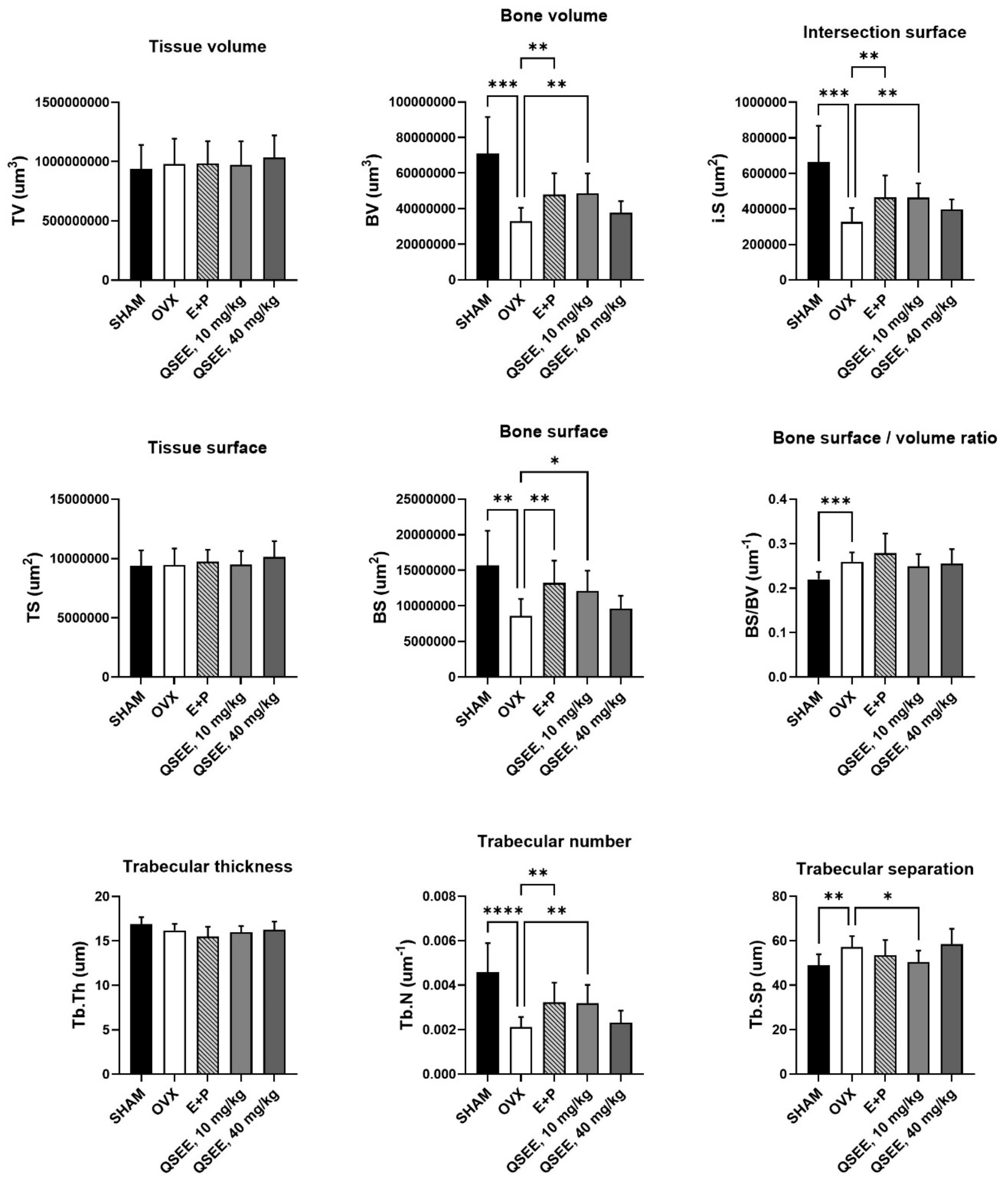

Supplementary Figure S3. Effects of QSEE treatment on bone parameters in ovariectomized mice

Compared to the OVX group, stars represent significant differences (* $p < 0.05$, **** $p < 0.0001$).

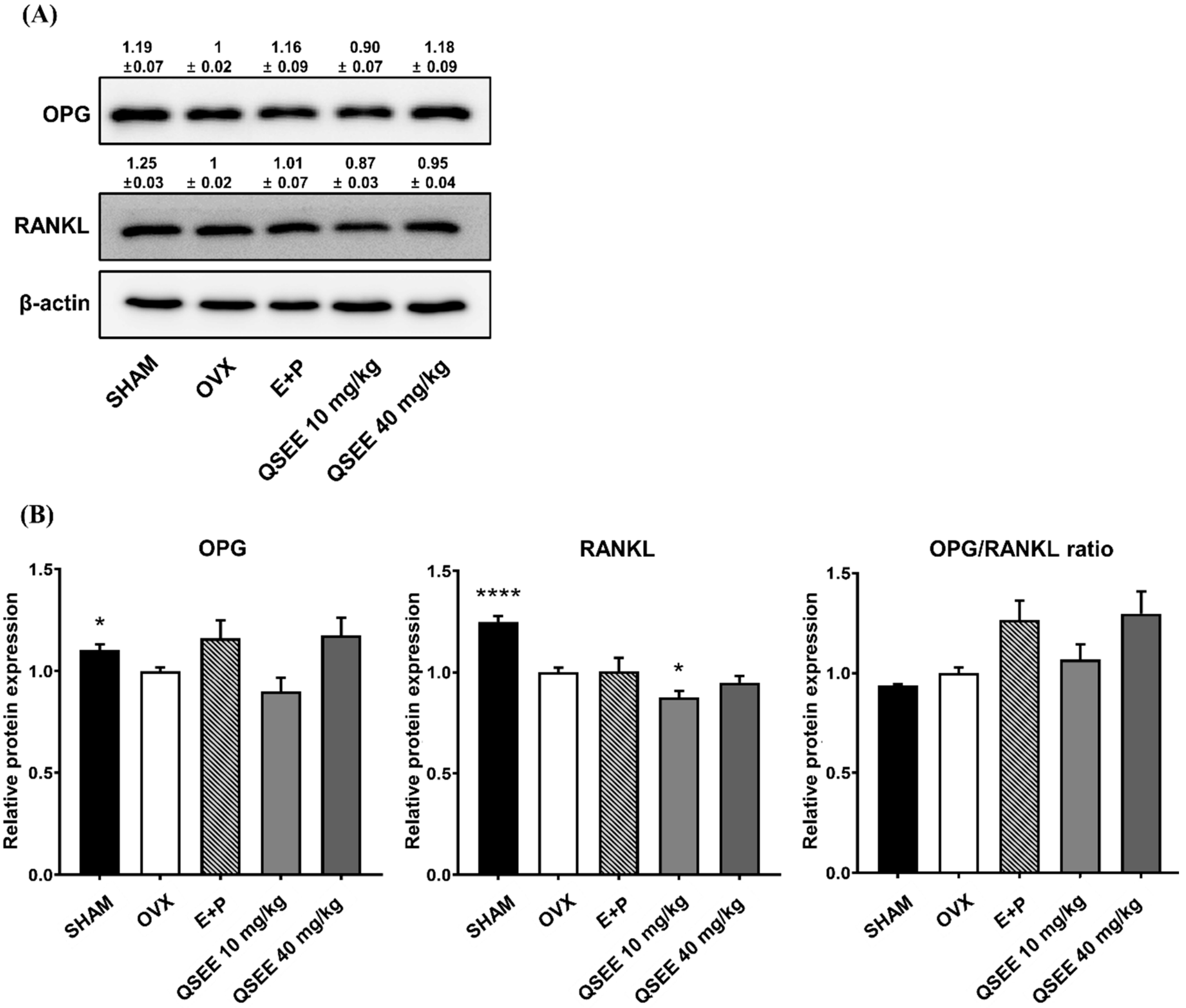

Supplementary Figure S4. OPG/RANKL ratio in primary cells (A) Western blot images for OPG and RANKL (B) Protein expression levels of OPG (C) RANKL (D) OPG/RANKL ratio. Compared to the OVX group, stars represent significant differences (* $p < 0.05$, ** $p < 0.01$, **** $p < 0.0001$).

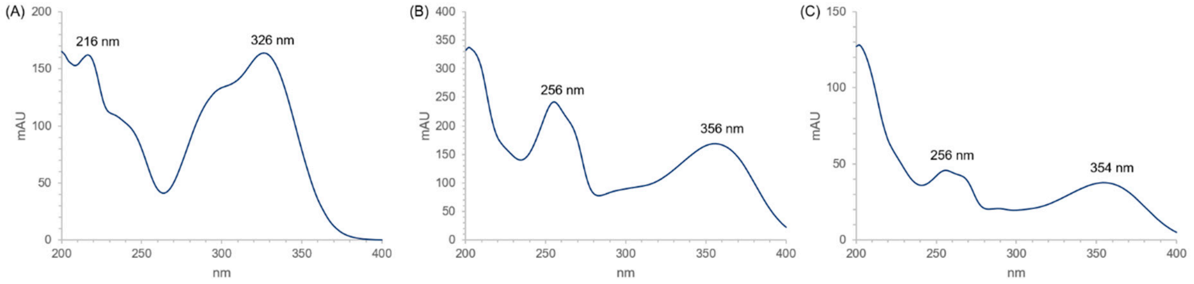

Supplementary Figure S5. **UV absorption spectra of the three major peaks detected in the HPLC chromatogram of QSEE.** (A) Peak 1, identified as chlorogenic acid; (B) Peak 2, putatively annotated as quercetin hexoside; and (C) Peak 3, putatively annotated as isorhamnetin hexoside
